# Harnessing Metal-Halide Layered Perovskite Structures for Next-Generation Lighting Sources

**DOI:** 10.1021/accountsmr.5c00246

**Published:** 2026-04-01

**Authors:** Balaji Dhanabalan, Milena P. Arciniegas

**Affiliations:** 121451 Automated Nanomaterials Engineering, Center for Convergent Technologies, Istituto Italiano di Tecnologia (IIT), Via Morego 30, 16163 Genoa, Italy

## Abstract

Advances in nanoscale semiconductor materials are enabling next-generation optoelectronic technologies with unprecedented efficiency, spectral control, and device miniaturization. Achieving this potential, however, requires the development of so-called *Triple E* materials, that is, systems that are environmentally friendly, economically inexpensive, and energetically efficient. Meeting these three criteria simultaneously remains a significant challenge. Metal-halide perovskites have emerged as remarkable semiconductors due to their strong optical absorption, high carrier mobility, long diffusion lengths, defect tolerance, and widely tunable bandgaps. Despite these outstanding properties, their limited ambient and operational stability continues to constrain their large-scale preparation and integration into robust devices.

Our research has focused on metal-halide layered perovskites, including Pb-free Sn-based analogues, as promising platforms to address these limitations. In these materials, alternating organic and inorganic layers form natural quantum wells that provide intrinsic electronic and dielectric confinement. This well-defined layered architecture enables tunable, broadband light emission from a single material component, without the need for multiple emissive layers. By avoiding complex multilayer architectures, device fabrication is simplified while interfacial defects, self-absorption effects, and differential degradation pathways are reduced. Moreover, the incorporation of bulky organic cations further enhances environmental stability by increasing hydrophobicity and protecting the inorganic framework from moisture.

Beyond structural protection, organic cations play an active role in defining the optoelectronic response. Their size, functionality, and conformation influence octahedral distortions, interlayer spacing, and exciton binding energies. Importantly, we have also shown that interactions between organic cations and solvents during synthesis can influence molecular conformation and octahedral connectivity, thereby directly modulating emission properties and charge transport. This solvent-cation interplay represents a largely unexplored avenue for structural and photophysical tuning.

In this Account, we expand upon these advances with a focus on Ruddlesden–Popper organic–inorganic layered perovskites and related structures as efficient and reconfigurable light emitters. We summarize synthetic and design strategies that exploit organic cation engineering and metal substitution to tailor emission across the visible spectrum while addressing toxicity concerns through partial or complete replacement of Pb. The inherent structural versatility of layered perovskites also allows their integration into flexible substrates, reversibly modulating their emission through postsynthetic treatments or mechanical stimuli, broadening their functional scope toward strain-controlled emission.

Looking forward, the convergence of artificial intelligence (AI), automated synthesis, and high-throughput characterization offers a transformative route to navigate the vast compositional and structural chemical space of organic–inorganic layered perovskites. By coupling data-driven discovery with mechanistic insight, it becomes possible to accelerate the identification of advanced, stable, efficient, and application-specific structures. Such an integrated approach will be essential to translating layered perovskites from promising laboratory materials to technologically viable platforms that fulfill the *Triple E* paradigm and enable the next generation of sustainable optoelectronic devices.

## Graphical abstract

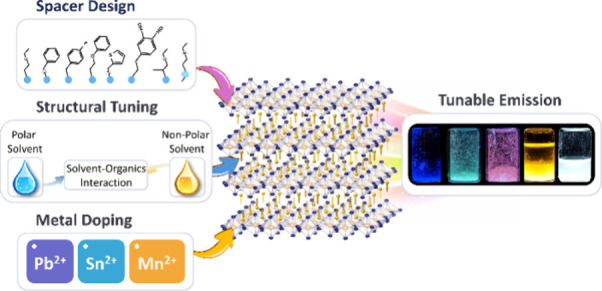

## 1 Introduction

Developing advanced semiconducting materials that combine high efficiency with long-term stability is central to modern optoelectronics.^1^ Beyond photovoltaics, high-performance devices such as light-emitting diodes (LEDs), lasers, and sensors increasingly demand low-cost, easily processable materials. At reduced dimensions, semiconductors offer unique opportunities to precisely control light-matter interactions.^2^ Yet achieving high efficiency, environmental stability, and scalable fabrication simultaneously remains a challenge.

Solution processability, tunable optical bandgaps, high color purity, and strong luminescence have positioned metal-halide perovskite nanocrystals as promising emitters for lighting. However, despite advances in passivation chemistry, interface engineering, and encapsulation strategies, limited environmental and operational stability continues to hinder commercialization.^3^ Alternative hybrid platforms, such as luminescent metal–organic frameworks (MOFs), offer modular structural tunability but are often limited by poor intrinsic conductivity and suboptimal optical performance.^4,5^

Low-dimensional organic–inorganic metal-halide layered perovskites are an attractive intermediate platform that combines structural tunability with exciton-driven light emission and low-temperature processability.^6^ These materials consist of alternating inorganic metal-halide layers and bulky organic cations (Figure 1), forming natural quantum wells that provide both quantum and dielectric confinement while enhancing environmental stability. Their general formula *L*_*m*_ *A*_*n*–1_ *M*_*n*_ *X*_3*n*+1_ defines composition, where *L* is a bulky organic cation, *A* a small monovalent cation (e.g., Cs^+^, MA^+^, FA^+^, with MA^+^ and FA^+^ referring to methylammonium and formamidinium, respectively), *M* a divalent metal cation (e.g., Pb^2+^, Sn^2+^, Cu^2+^, and Ge^2+^), and *X* a halide anion (Cl^–^, Br^–^, I^–^); *m* represents the type of organic cation, such as monocations (*m* = 1) or dications (*m* = 2) and *n* the number of metal-halide layers. Varying *n* enables a transition from purely 2D (*n* = 1) to quasi-2D layered perovskites (*n* > 1), allowing systematic control over electronic confinement and emission properties.

**Figure 1:**
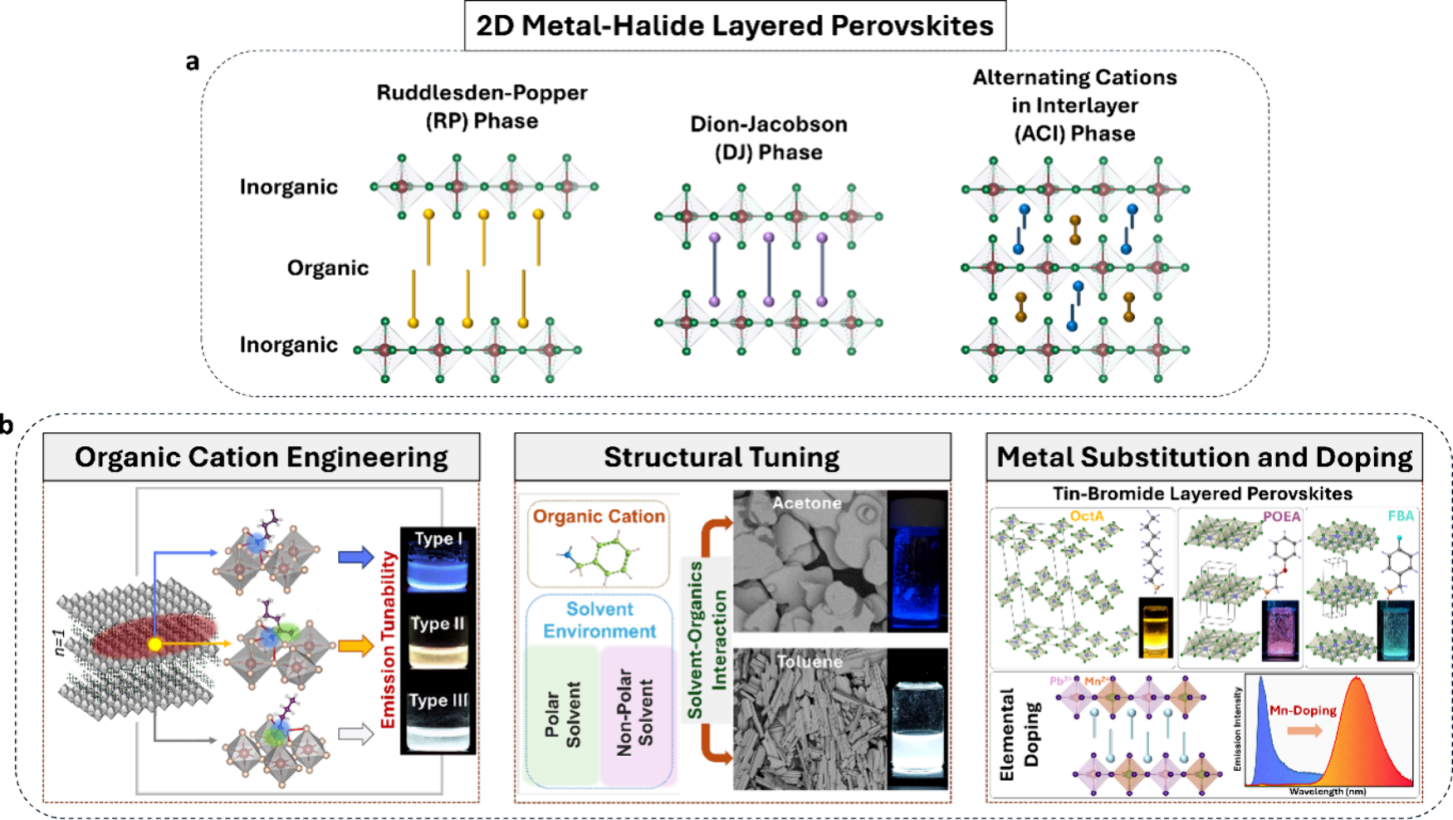
**a**, Structural types of 2D layered perovskites: RP perovskites with monoammonium organic cations, DJ with diammonium cations, and ACI with similarly sized cations. **b**, Our strategies to tune the photophysical properties of RP layered perovskite structures for advanced lighting applications: Organic cation engineering. Adapted with permission from reference^10^. Copyright 2021 Wiley-VCH; structural tuning. Adapted with permission from reference^11^. Copyright 2025 Wiley-VCH; and metal substitution and doping. Adapted with permission from reference^12^. Copyright 2023 American Chemical Society.

The choice of organic cation determines whether the layered perovskite adopts a Ruddlesden–Popper (RP), Dion-Jacobson (DJ), or alternating cation interlayer (ACI) structure. RP phases are the most extensively studied due to their structural simplicity and well-defined van der Waals gap. DJ structures eliminate this gap through divalent organic cations, often enhancing structural integrity and out-of-plane charge transport,^7^ although increased rigidity can limit lattice flexibility and phase purity.^8,9^ ACI structures attempt to combine the advantages of both architectures but remain comparatively less explored.

The structural versatility of layered perovskites translates directly into tunable emission. Consolidated strategies such as halide mixing, phase-transition control, and the formation of quasi-2D structures allow bandgap engineering.^13^ The electronic structure of metal-halide perovskites arises from antibonding interactions between halide *p* orbitals and metal *s* orbitals,^14^ enabling straightforward modulation through compositional and structural design. Increasing the number of inorganic layers (*n*) reduces quantum confinement and narrows the bandgap, though achieving phase-pure quasi-2D systems remains challenging.

Beyond inorganic composition, bulky organic cations play a decisive role in determining exciton dynamics and emission efficiency. By inducing lattice distortions and modulating dielectric confinement, organic cations influence exciton binding energies, electron–phonon coupling, and the formation of self-trapped excitons (STEs) or defects.^15^ In parallel, metal substitution and elemental doping provide complementary tools to regulate the inorganic framework, influencing electronic structure and defect tolerance. Together, organic layer engineering and metal-site modifications constitute two interdependent design dimensions for tailoring emission in layered perovskites.

In this *Account*, we focus on purely 2D (*n* = 1) RP Br-based layered perovskites and related structures, demonstrating how organic cation design and metal site modification can be harnessed to modulate emission properties (Figure 1b). We show how molecular architecture, including conformation, steric effects, and conjugation, governs lattice dynamics and exciton localization, and how metal substitution and controlled doping enable both toxicity reduction and emission tunability. Through this synergistic approach, emission can be tuned from narrowband to broadband and white-light regimes within a single-component layered system, providing practical guidelines for rational design of stable, tunable, and high-performance layered structures for next-generation optoelectronic applications.

## 2 Organic Cation Engineering Strategies in RP Layered Perovskites

Organic cations play a central structural and functional role in 2D layered perovskites. Although they do not directly contribute to the band edge electronic states, their molecular configuration, size, and electronic characteristics strongly modulate the inorganic framework and its optoelectronic properties.^16^ By separating the metal-halide layers, organic cations define natural quantum-well architectures that confine charge carriers and excitons within the inorganic layer, thereby affecting band alignment, dielectric screening, excitonic behavior, and interfacial electronic coupling. As a result, rational organic design has emerged as a powerful strategy to finely tune the photophysical properties, phase stability, and emission characteristics of RP layered perovskites and related low-dimensional structures.^12,17^

Building on this understanding, our team has focused on coupling organic cation engineering with sustainable material processing by initially developing a *Triple E* (environmentally friendly, economically inexpensive, and energetically efficient) room-temperature synthesis (Figure 2a). Using acetone as a more eco-friendly polar solvent compared to other alternatives such as DMSO and DMF, to prepare deep-blue-emitting RP layered perovskite platelets (Figure 2b),^18^ we investigated alkylamines with different aliphatic chain lengths (butylammonium, BA; octylammonium, OctA; and decylammonium, DA) and arylalkylamines (benzylammonium, BzA; dopammonium, DopA; and phenethylammonium, PEA). We found that the PEA-based crystals exhibited a higher blue photoluminescence quantum yield (PLQY) compared to BA-based crystals, while the structures prepared with organic cations such as OctA- and DA displayed bluish-white emission yet poor PLQY after exfoliation. An earlier study by Sargent and co-workers^17^ showed that BA-based single crystals exhibited larger displacements of Br and Pb atoms, indicating strong phonon activation and coupling to electronic excitations. Such enhanced vibrational overlap between the ground- and excited-state wave functions promotes faster nonradiative decay.

**Figure 2:**
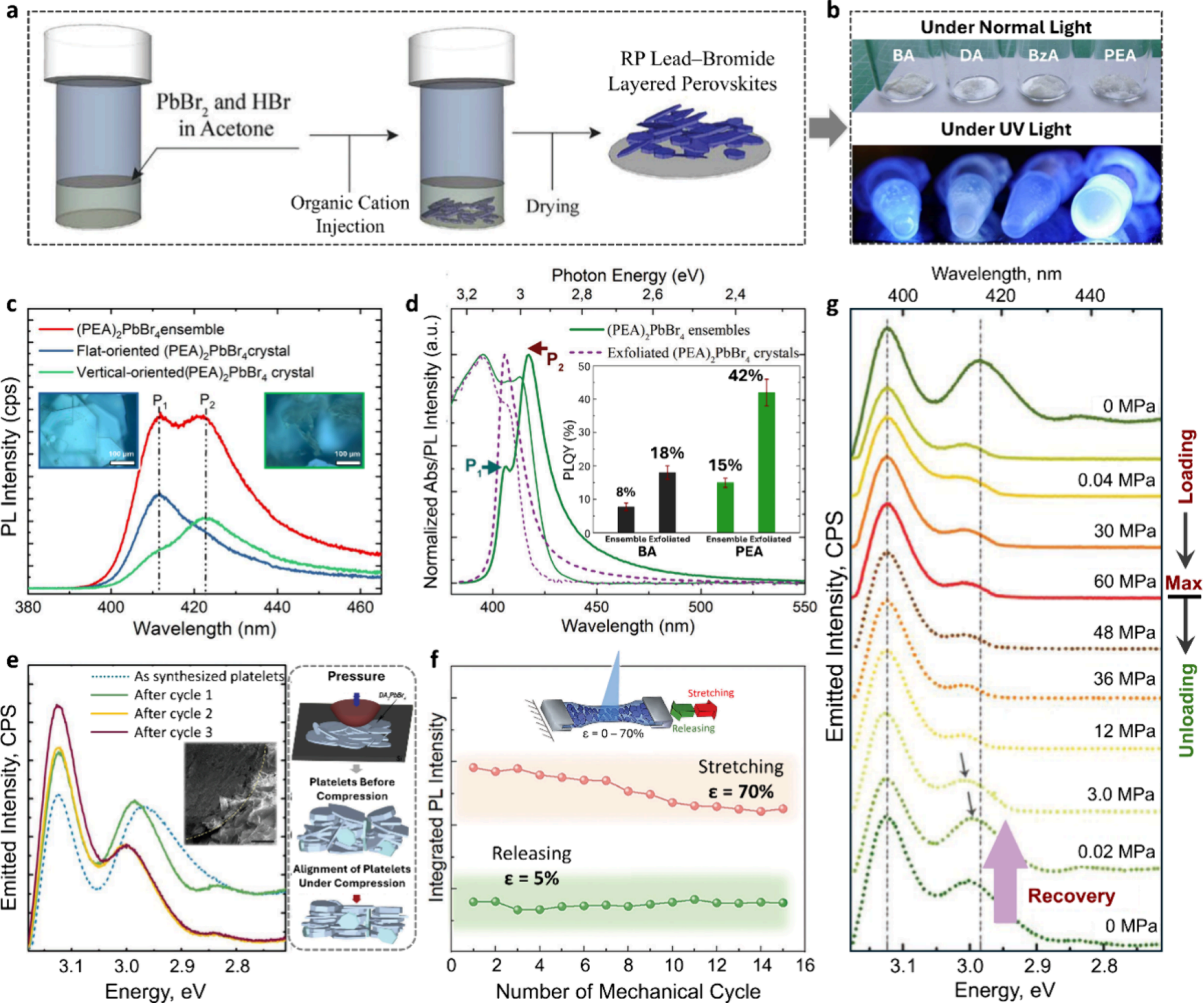
**a**, Room-temperature synthesis of RP layered perovskites. **b**, Photographs of RP platelets under ambient and UV illumination. **c**, Micro-PL spectra of PEA-based platelets with different orientations. **d**, PL and absorption spectra before and after exfoliation. Inset: Average PLQY of BA- and PEA-based crystals. Adapted from reference^18^. Copyright 2019 Royal Society of Chemistry. **e**, PL evolution of DA-based crystals under cyclic compression. Adapted with permission from reference^19^. Copyright 2019 Wiley-VCH. **f**, PL intensity of BzA-based polymer composites under cyclic tensile stress. Adapted from reference^20^. Copyright 2021 Royal Society of Chemistry. **g**, In-situ PL of DA-based platelets during cyclic loading. Adapted with permission from reference^19^. Copyright 2019 Wiley-VCH.

By contrast, the more rigid structure of PEA-based systems suppresses such coupling, resulting in comparatively higher PLQY, as also supported by other experimental and theoretical studies.^21^ However, the overall structural rigidity of layered perovskites is dictated by multiple interdependent factors, including the intrinsic rigidity or flexibility of the organic cation, its steric profile, hydrogen-bonding motifs, and the presence of dipoles or heteroatoms, which determine how the organic cation couples to and perturbs the inorganic framework, influencing both in-plane and out-of-plane octahedral distortions and lattice connectivity.

Beyond the intrinsic role of organic cations, the orientation of RP layered perovskite platelets relative to the substrate critically influences emission behavior. In PEA-based RP platelets, micro-PL mapping revealed pronounced orientation-dependent PL modulation (Figure 2c): flat-oriented platelets exhibited a strong high-energy emission contribution, whereas regions dominated by vertically oriented crystals showed enhanced low-energy emission,^18^ consistent with how optical path length and reabsorption effects vary with platelet orientation. By mechanically exfoliating the crystals, their orientation is constrained to be strictly parallel to the substrate, effectively suppressing reabsorption effects (Figure 2d). In these exfoliated platelets, reabsorption becomes largely confined to a single layer and is further reduced by the orthogonality of exciton wave functions; carriers with out-of-plane wave functions experience a higher effective bandgap than their in-plane counterparts, creating an energetic barrier that limits exciton relaxation to the lowest band-edge state. As a result, exfoliated crystals exhibit up to a 3-fold enhancement in PLQY compared to ensemble samples (inset in Figure 2d), in line with other reports where mechanical exfoliation of layered perovskite flakes yields a suppression of low-energy emission and a marked increase in PLQY.^22^

Earlier, we established that mechanical stress can actively modulate the orientation of layered perovskites and thus their emission. In DA-based layered perovskite crystals, cyclic mechanical loading in the megapascal range (up to 60 MPa) induced preferential platelet orientation without structural damage, enabling reversible PL modulation (Figure 2e, g).^19^ The same orientation-mediated mechanism applies to free-standing polymer composites prepared through in situ crystallization of BzA-based layered perovskites within a stretchable, free-standing polydimethylsiloxane (PDMS) under tensile stress of less than 2 MPa. We observed a ca. 2.5-fold increase in emission intensity (Figure 2f) for 70% strain, with spectral shifts that are fully reversible upon stress release.^20^ Although many reports in the literature demonstrate that external pressure or strain can substantially alter the PL of layered perovskites,^23^ such effects are often observed at high pressure (in the gigapascal range) in static diamond anvil cells. Strain-engineering studies further show that even small biaxial strain of less than 1% can alter the PL spectrum via octahedral tilting and lattice expansion, albeit typically at low temperature or through substrate-imposed strain.^24^ Achieving significant and reversible PL modulation at megapascal pressure is crucial for applications in soft optoelectronics and robotics, and flexible displays, where devices must operate under gentle mechanical deformation rather than extreme compression. Mechanically induced enhancements in PL intensity and spectral tunability under low and cyclic stress, as enabled by platelet orientation, therefore represent a particularly promising route toward practical strain-responsive perovskite emitters with dynamic control over light emission.

### Organic Cations Inducing Broadband Emission

The structural versatility of RP layered perovskites enables the incorporation of a wide range of organic cations whose configuration and molecular characteristics directly influence key structural parameters such as octahedral distortions, bond angles, rotation, and metal-halide bond lengths within the inorganic layer. These localized lattice distortions strongly impact the electronic band structure and favor the formation of STEs. The associated strong electron–phonon coupling in the distorted lattices induces transient lattice deformations that facilitate radiative recombination via sub-bandgap states, enabling broadband emission across the visible spectrum at room temperature. The magnitude and nature of these distortions, and thus the emission characteristics, are determined by molecular descriptors of the organic cations (so-called ‘*spacers*’), including molecular length, hydrogen-bonding, steric hindrance, bulkiness, and the chemical nature of the binding headgroup. A comprehensive review of spacer cations engineering and applications was provided by Kanatzidi’s group in 2021,^25^ highlighting the critical role of organic cations in directing structure–property relationships.

Despite this progress, the vast chemical space of potential organic cations makes it challenging to identify molecular features that promote broadband emission. Many studies to date have relied on randomly selected organic cations, with PEA often used as a model organic cation for structures showing narrow deep-blue emission rather than a broadband behavior.^17^ Recent efforts have begun to screen organic cation libraries in a more targeted way; for example, alkylammonium cations with increased steric bulk or multifunctional head groups have been shown to enhance STE formation and spectral breadth in Pb–I and Pb–Br layered perovskites.^26^ In our design strategy for broadband-emitting RP Pb–Br perovskites (Figure 3a),^10^ we focused on two complementary molecular descriptors: (i) the local structure of the binding group, which determines orientational freedom at the anchoring site and influences hydrogen-bonding interactions with the inorganic layer, and (ii) the overall molecular length, which influences structural rigidity and steric constraints.

**Figure 3:**
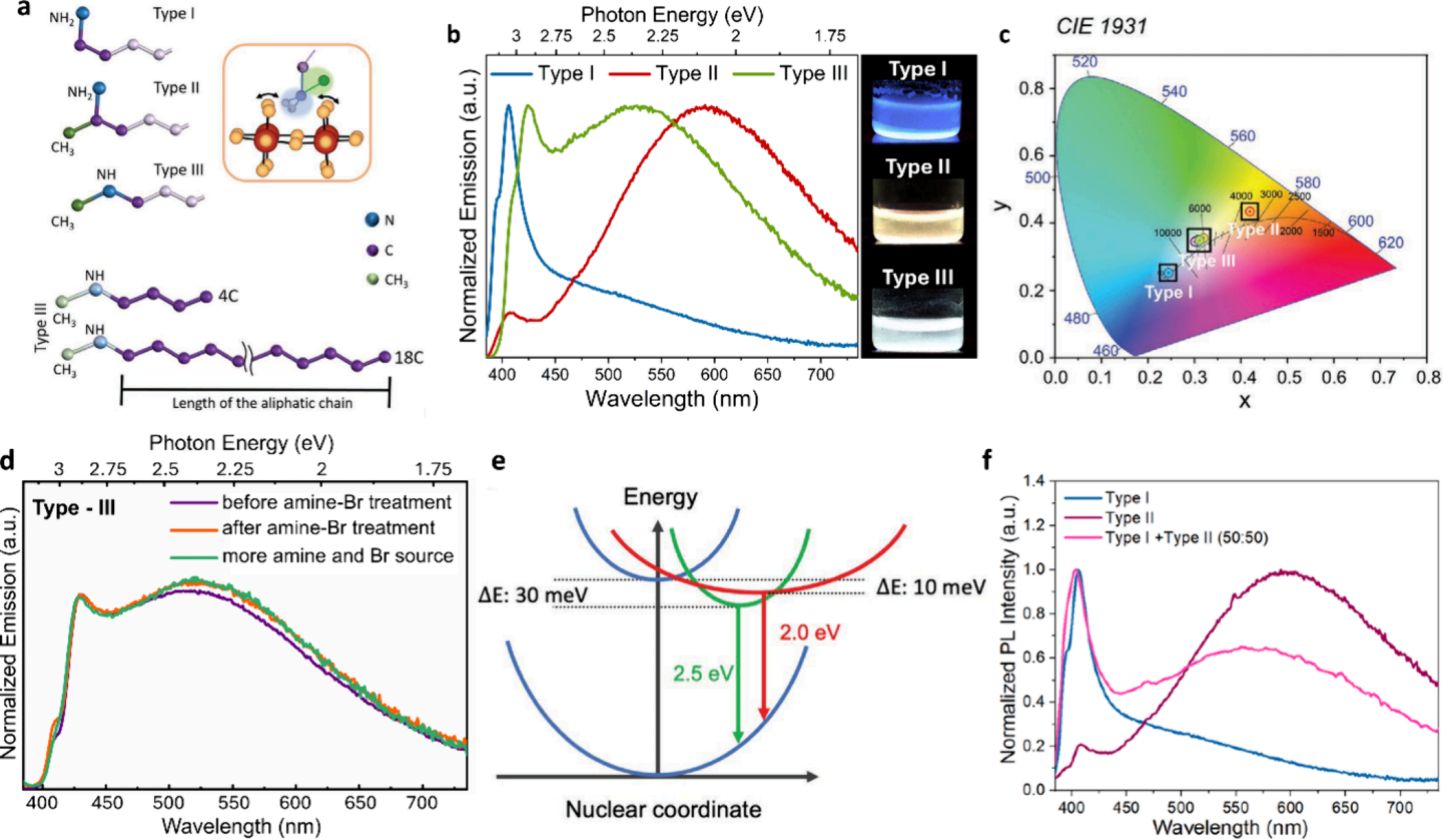
**a**, Molecular structures of three organoammonium cation head groups. Bottom: Type III amines with aliphatic chains (C4 to C18). Inset: cation anchoring between four Pb–Br octahedra. **b**, PL spectra and UV photographs of perovskite crystals prepared with the three cation types. **c**, Corresponding CIE color coordinates. **d**, PL spectra of type-III crystals upon amine and bromide post-treatments. Adapted from reference^10^. Copyright 2021 Wiley-VCH. **e**, Energy landscape of trapping/detrapping and emission pathways from temperature-dependent PL measurements. Reproduced from reference^27^. Copyright 2022 Wiley-VCH. **f**, PL spectra of crystals synthesized using a 50:50 mix of type I and type II cations. Adapted from reference^10^. Copyright 2021 Wiley-VCH.

To probe binding group effects, we selected three representative organoammoniums classes: Type I – primary ammonium with linear chains (e.g., undecylammonium, UDA); Type II – primary ammonium with branched chains, where a methyl group is directly bonded to the carbon adjacent to the ammonium group (e.g., 1-methyldecylammonium, 1-MDA); and Type III – secondary ammonium with one methyl and one linear chain (e.g *N*-methyldecylammonium, N-MDA).

RP layered perovskite structures incorporating longer alkyl chains exhibited slightly larger optical bandgaps than those with shorter chains, consistent with increased spacing between inorganic layers and weaker interlayer electronic coupling. Compared with the type I crystals, which showed narrow deep-blue emission dominated by free excitons (Figure 3b), type II organic cations introduced greater structural distortions that suppressed free-exciton emission and favored broadband STE emission. Type III crystals displayed dual emission, with a narrow deep-blue free-exciton peak alongside a broadband STE component, resulting in spectrally balanced white-light emission (Figure 3b-c). This broadband STE behavior originates from intraoctahedral distortions induced by the methyl substituent adjacent to the ammonium group, which modifies hydrogen-bonding interactions and reorients the organic cation relative to the inorganic layer.

These results highlight the key role of the organic cation binding group in tuning emission pathways. In contrast to many prior studies on 2D layered perovskites in which only chain length or lattice dimensionality was varied,^8^ our work disentangles binding group effects from overall chain length. While aliphatic chain length had minimal influence on the emission spectral profile of the Type III white-emitting crystals, it strongly impacted emission intensity and PLQY through its effects on surface passivation and structural rigidity conferred by the organic layer. This observation aligns with recent reports in layered perovskites showing that long-chain, bulky, or multifunctional organic cations can enhance PLQY by suppressing nonradiative recombination and defect-related losses.^28^

While broadband emission in RP layered perovskites can also originate from intrinsic defect states such as halide or organic vacancies,^29^ which create sub-bandgap emission centers, our fluence-dependent PL studies indicate that the broadband emission in type III crystals is intrinsic in nature and dominated by STE processes, as confirmed by linear intensity scaling and insensitivity to postsynthetic passivation treatments. In contrast to the defect-mediated broadband emission reported by Yin et al.,^29^ the broadband emission in our systems persisted upon amine and bromide post-treatments (Figure 3d), supporting its STE origin. Detrapping barriers for STE emission extracted from temperature-dependent PL measurements are shown in Figure 3e. Motivated by these results, we further explored the coincorporation of complementary organic cations to combine distinct emission pathways. A 1:1 mixture of type I and type II cations produced RP layered perovskite crystals that simultaneously show a narrow blue and a broadband component, yielding intrinsic white-light emission (Figure 3f). This strategy parallels recent efforts in layered perovskite heterointerfaces and mixed spacer systems, where combining cations with distinct steric and electronic characteristics enables multimodal emission and spectral broadening desirable for lighting and display applications.^30^

### Conjugated Organic Cations and Reversible Emission Tunability

Beyond structural distortions, organic cations are a tool for tuning energy-level alignment at the organic–inorganic interface in RP layered perovskites. By modifying the highest occupied and the lowest unoccupied molecular orbital (HOMO/LUMO) levels of the organic layer, conjugated cations can actively regulate interfacial charge transfer, exciton localization, and radiative recombination pathways. A clear demonstration of this approach was reported by Gao and co-workers^31^ who used a family of conjugated monoammonium cations incorporating thiophene π-units, sterically hindered methyl groups, and strongly electron-withdrawing cyano groups. The tailored molecular conformation and electronic structure of these organic cations enabled the formation of single-crystalline quantum wells with efficient interfacial energy transfer from the inorganic layer to the organic layer, resulting in tunable emission from green to red. Closely related approaches exploit functional groups that impose directional intermolecular interactions within the organic layer. For example, introducing carboxylic acid (−COOH) functional groups into organic cations promotes the formation of one-dimensional hydrogen-bonded networks, which break in-plane symmetry and drive the growth of layered perovskite nanowires.^32^ In addition to modulating emission, π-conjugated organic cations can enhance charge transport in RP layered perovskites. Electron-rich aromatic units such as thiophene introduce a highly polarizable sulfur atom and loosely bound π-electrons, contributing additional near-band-edge states that facilitate carrier delocalization. In another example, Ni. C et al.,^33^ incorporated 2-thiophenemethylammonium (TMA) as an organic cation in RP layered perovskites, leading to a modification of the electronic structure and power conversion efficiencies approaching 19% in photovoltaic devices.

Guided by these advances, our team further investigated the role of thiophene-containing organic cations in modulating the emission characteristics of RP layered perovskites.^34^ Leveraging the versatility of our *Triple E* synthesis route, we fabricated thiophene-based RP layered Pb-bromide perovskite platelets using TMA and 2-thiopheneethylammonium (TEA) as organic cations. This approach enables control over crystal morphology, from platelet-like to elongated structures, by varying both the identity of the thiophene cation and the HBr concentration during synthesis (Figure 4a). Instead, TEA-based RP layered perovskites consistently exhibited platelet-like morphologies, regardless of variations in organic cation or HBr concentration, highlighting the strong structure-directing influence of the organic cation. These morphological variations translated directly into distinct emission behaviors. Platelet-like crystals exhibited deep-blue emission with weak broadband features, plausibly arising from crystal defects such as organic and halide vacancies. In TEA-based crystals (Figure 4b), increasing the organic and halide concentration effectively suppressed the broadband emission, further supporting a defect-related origin. By contrast, elongated structures displayed comparably intense narrow deep-blue and broadband emission. This dual emission is attributed to the coexistence of free excitons and efficient STEs (Figure 4c).

**Figure 4:**
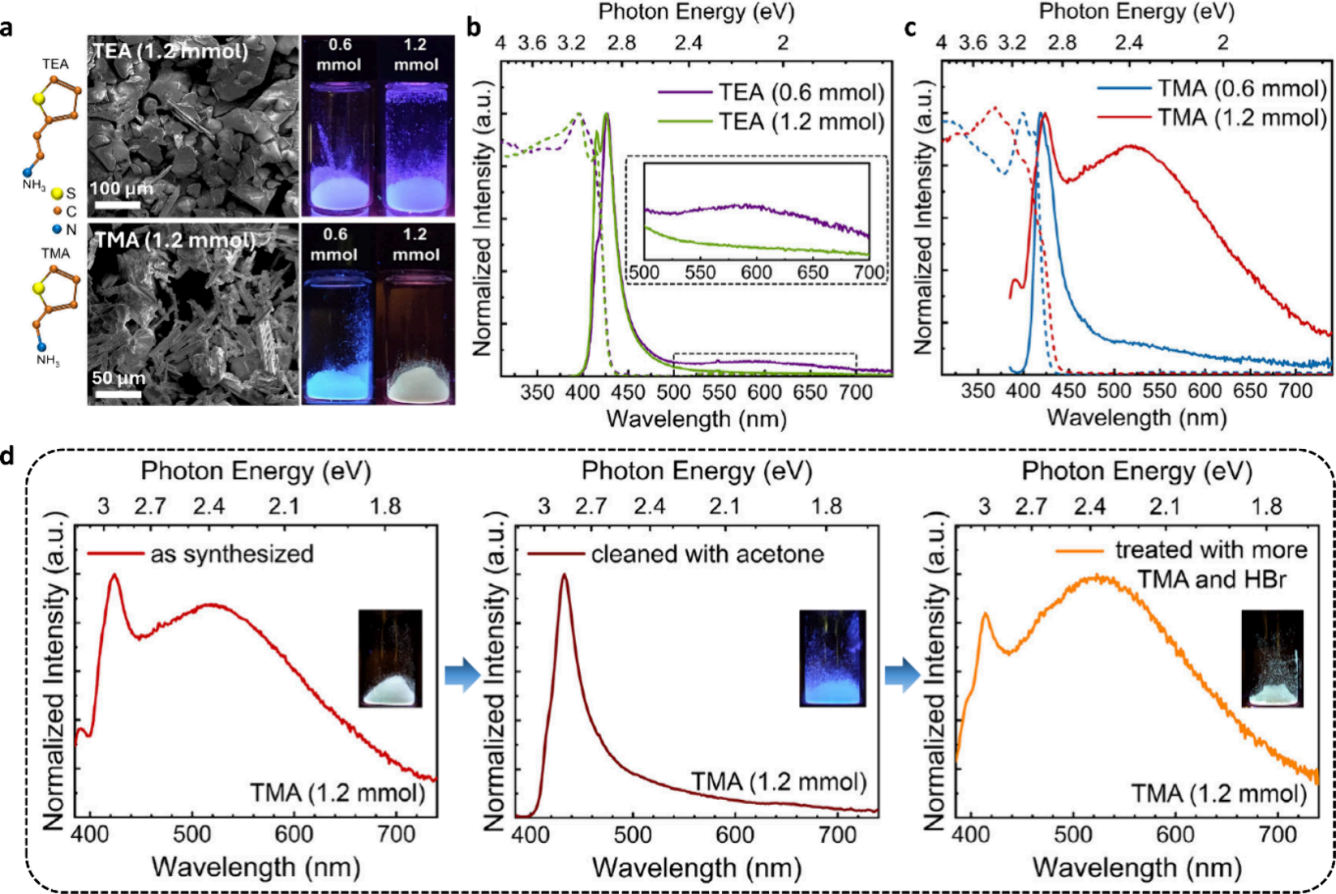
**a**, SEM images of RP perovskite crystals from the *Triple E* synthesis route with TEA and TMA organic cations at varying HBr concentration. Left: molecular structures; right: photographs of the crystals under UV light. **b, c**, Absorption (dashed lines) and PL (solid lines) spectra of TEA- and TMA-based crystals with stoichiometric (0.6 mmol) or excess (1.2 mmol) organic cation/halides. Inset: weak broadband in TEA-based crystals suppressed with excess precursors. **d**, Reversible emission switching: white (left) to blue (after washing) to white (after TMA-Br treatment) over multiple cycles. Inset: corresponding UV photographs of the crystals. Adapted from reference^34^. Copyright 2021 Wiley-VCH.

We then explored postsynthetic strategies to demonstrate reversible emission switching in TMA-based crystals (Figure 4d). Washing white-emitting elongated crystals with acetone transformed them into deep-blue emissive platelets, likely due to the removal of excess organic cations and concomitant structural reorganization. This interpretation is consistent with prior reports showing that coordinating solvents such as acetone, DMF, and DMSO can interact with the metal-halide framework and modify local octahedral connectivity.^35^

Subsequent treatment with precursor solutions containing organic cations and halides restored both the elongated morphology and white-light emission, enabling multiple reversible cycles. In contrast, washing with toluene produced no observable changes, suggesting that acetone can penetrate the lattice and organic vacancies, thereby facilitating structural rearrangement. Similar solvent- and post-treatment-mediated tuning of emission has been reported in layered perovskites, where coordinating solvents, antisolvent washes, or ligand/halide treatments modulate defect populations, exciton trapping, and crystallization pathways, leading to enhanced or reconfigured PL.^35^ These studies support our observation that solvent interactions and postsynthetic processing can dynamically control structural motifs and emissive pathways, enabling reversible emission switching in 2D RP perovskites. These strategies enable spectrally tunable, reconfigurable optoelectronic materials.

### Solvent-Organic Cation Interactions and Structural Tunability

Solvent-induced structural transformations provide an additional and powerful dimension for tuning RP layered perovskites beyond intrinsic organic-cation design. A notable example was reported by Kanatzidis and co-workers,^36^ who used propylammonium organic cation to induce a transition from electronically 1D “stepped” perovskite layers, containing both corner-sharing and face-sharing octahedra, to electronically 2D, flat RP phases.

Inspired by these considerations, we extended our *Triple E* synthesis route to a range of polar and nonpolar solvents (Figure 5a) to probe solvent-organic cation interactions. Two representative organic cations were examined: flexible aliphatic BA and more rigid, π-conjugated BzA, enabling isolation of the combined effects of solvent polarity and cation structure on crystal growth. BzA-based RP perovskites crystallized into conventional platelets in polar solvents such as acetonitrile and acetone. In contrast, nonpolar solvents such as toluene and benzene yielded elongated crystals with periodic nanoscale grooves (Figure 5b), resembling morphologies observed in TMA-based systems (Figure 4a). Crystallographic analysis revealed that such groovy structures form low-symmetry ribbon-like substructures composed of disconnected corner-sharing [Pb_3_Br_13_]_n_ octahedral segments (Figure 5c). This disrupted connectivity enhances quantum confinement, evidenced by sharper absorption features at ∼ 3.29 and 3.14 eV (Figure 5d). However, the mechanistic origins of these grooved morphologies and strategies for their precise control remain an open question.

**Figure 5:**
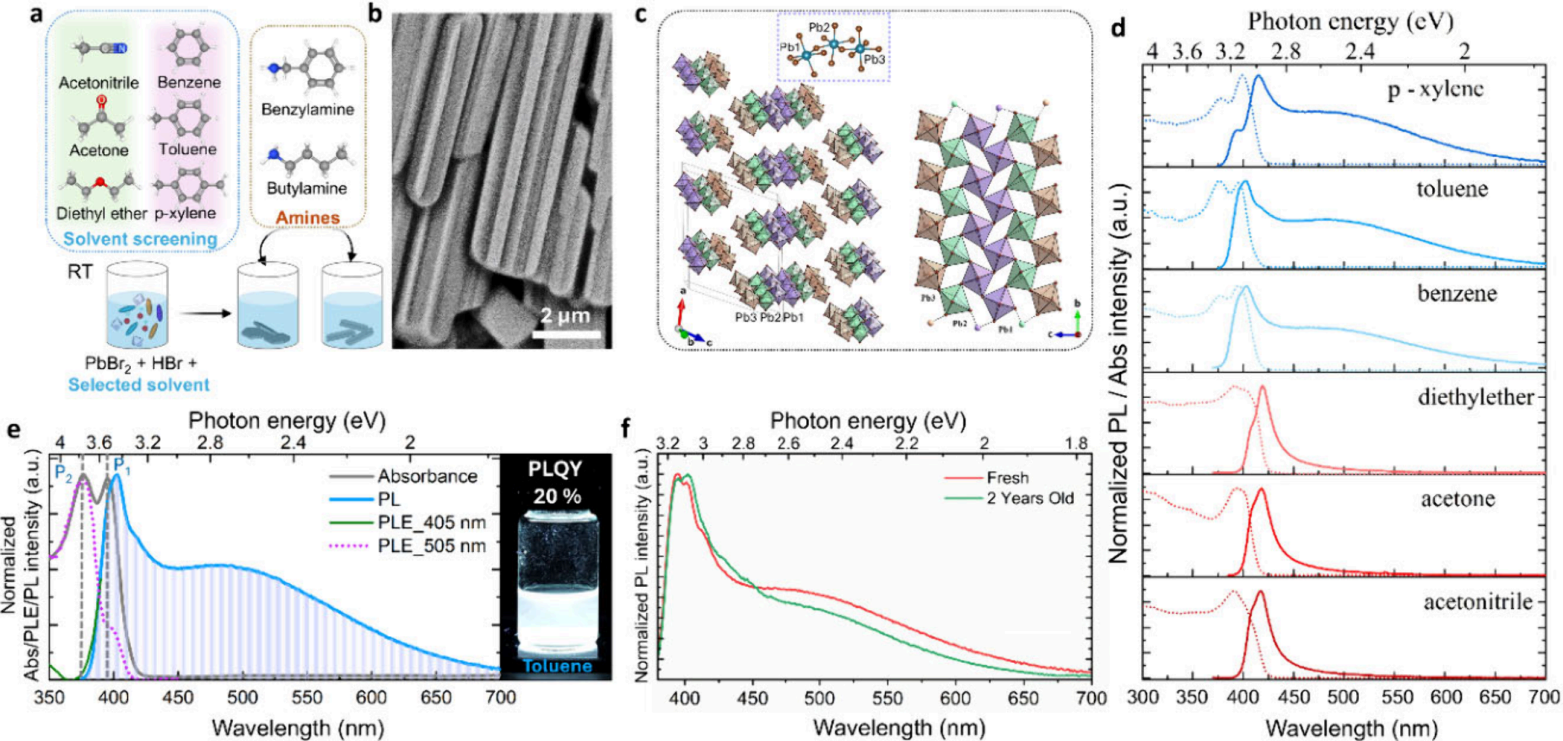
**a**, Molecular structures and schematic of the *Triple E* synthesis route for studying solvent-organic cation interactions. **b**, SEM image of BzA-based RP-related perovskite crystals prepared in toluene showing nanoscale periodic grooves. **c**, Crystal structure from 3D electron diffraction (3D ED) analysis showing ribbon-like, disconnected inorganic octahedra. The dotted box highlights the connectivity of three independent Pb atoms. Right: expanded [Pb_3_Br_13_]_n_ ribbon oriented along the *b*-axis. **d**, Absorption (dotted lines) and PL (solid lines) spectra of BzA-based RP and RP-related perovskite crystals in different solvents. **e**, Absorption (gray), PL excitation (pink and green), and PL (blue) spectra of BzA-based crystals in toluene. P_1_ and P_2_ indicate the low- and high-energy absorption peaks. Right: photographs of the crystals under UV light. **f**, PL spectra of fresh and aged BzA-based crystals prepared in toluene and stored under ambient conditions. Adapted from reference^11^. Copyright 2025 Wiley-VCH.

Optically, these grooved BzA-based crystals display dual emission consisting of a narrow free-excitonic peak yielding white-light output (Figure 5e). With a PLQY of ∼ 18% and CRI values of ≈81, attributed to reduced electron–phonon coupling in the disconnected octahedral framework, these structures remain stable under ambient conditions for over two years (Figure 5f) with PLQY ∼ 7%, a gradual efficiency loss linked to edge-induced nonradiative recombination.

Further optimization of crystal morphology through solvent selection, alongside targeted edge-defect passivation strategies, could enhance PLQY and thermal stability. Integration of these groovy structures into polymer matrices or thin films may enable scalable, flexible, and durable white-light emitters. Beyond solid-state lighting, a deeper understanding of solvent-cation interactions opens opportunities for fabricating diverse architectures, including nanotubes, photonic crystals, and optical waveguides to engineer light-matter interactions at the nanoscale.

The approaches discussed in this section demonstrate that organic-cation engineering, in concert with solvent control, provides a powerful toolkit for manipulating lattice distortions, exciton–phonon coupling, and defect landscapes in 2D Pb-bromide layered perovskites, enabling broad tunability of emission properties. However, despite these advances, the continued reliance on Pb remains a fundamental limitation for practical deployment, motivating the exploration of Pb-free alternatives. Importantly, the structure–property relationships elucidated here, such as the regulation of octahedral connectivity, lattice rigidity vs softness, and interlayer interactions mediated by tailored organic cations, may guide the design of Pb-free systems. These insights naturally extend to the next frontier of materials development, where metal cation substitution and doping offer complementary routes to reduce toxicity, tune electronic structure, and further expand the functional landscape of layered perovskite systems.

## 3 Metal Substitution and Elemental Doping in RP Layered Perovskites

While organic cations primarily control interlayer interactions, lattice distortions, and exciton localization, modifying the inorganic layer composition directly tunes octahedral connectivity, electronic structure, and lattice softness, which are parameters that govern exciton formation, self-trapping, and radiative recombination. Metal-site engineering offers a strategy to reduce Pb content and design environmentally benign layered perovskites without sacrificing emission performance. By incorporating dopant or substituent metal cations and pairing them with tailored organic cations, it is possible to achieve synergistic control over lattice dynamics and excitonic processes, enabling precise tuning of band structure, emission characteristics, and photophysical behavior. This combined strategy provides a versatile pathway to stable, Pb-reduced or Pb-free RP perovskites with engineered light-emission properties, which we discuss in the following subsections.

### Metal Substitution

In the case of complete substitution, considerable attention has focused on replacing Pb^2+^ due to environmental and regulatory concerns associated with Pb toxicity. Isovalent substitution with less toxic metals from group 14, such as Sn^2+^ and Ge^2+^, as well as divalent transition metals (e.g., Zn^2+^, Cu^2+^, Fe^2+^, and Pd^2+^), has also been explored.^37^ Among these, Sn-based RP layered perovskites are considered the most promising Pb-free alternatives. These materials typically exhibit red-shifted emission profiles relative to Pb-based analogues, reflecting narrower bandgaps.^38^ This behavior arises from stronger Sn^2+^ *s* and halide *p* orbital hybridization, enhanced spin–orbit coupling, and altered valence band energetics.

Despite their promise, Sn-based systems face significant challenges, including suboptimal optical performance and limited operational stability. These limitations are primarily associated with high defect densities due to low defect formation energies, which generate undercoordinated Sn and halide ions, as well as spontaneous oxidation of Sn^2+^ to Sn^4+^ at unpassivated surfaces. The lower oxidation potential of Sn^2+^ compared to Pb^2+^ exacerbates this instability. Strategies to mitigate these issues include the use of additives, strongly coordinating solvents, and organic cations capable of forming strong hydrogen-bonding interactions with the inorganic layers. For example, incorporation of conjugated semiconducting or hydrophobic organic cations has been shown to improve crystallinity, enhance intrinsic stability, and reduce defect-assisted nonradiative recombination.^39^

Specifically, PEA-based Sn-halide perovskites exhibit increased structural rigidity relative to alkyl-chain organoammonium analogues, thereby suppressing Sn^2+^ oxidation and improving emission efficiency.^40^ Enhanced emission has also been reported for systems incorporating thienylethylammonium and 4-bromobenzylammonium cations,^41,42^ while cyanuric acid in TEA-based Sn–I systems prevented Sn^2+^ oxidation, yielding a 4-fold improvement in device performance.^43^ Comprehensive reviews summarizing Sn-halide perovskites for lighting applications have been recently reported.^44^

Extending these insights, our group developed a simple, low-cost, low-temperature synthesis protocol to fabricate stable, and efficient Sn-bromide RP layered perovskites using different organic cations (Figure 6a, b).^12^ Incorporation of octylammonium (OctA) cations produced thin, stable platelet-like crystals (Figure 6c). We identified toluene as an effective low-temperature solvent that interfaces with precursor mixtures containing SnBr_2_, HBr, and H_3_PO_2_, stabilizing the Sn-based crystals against oxidation while promoting fast crystal growth at ∼ 4 °C. The resulting OctA-based RP-related Sn–Br layered perovskite crystals exhibited a broadband yellow-orange emission, with a PLQY exceeding 80% and a long decay lifetime (∼3.2 μs) (Figure 6d).

**Figure 6:**
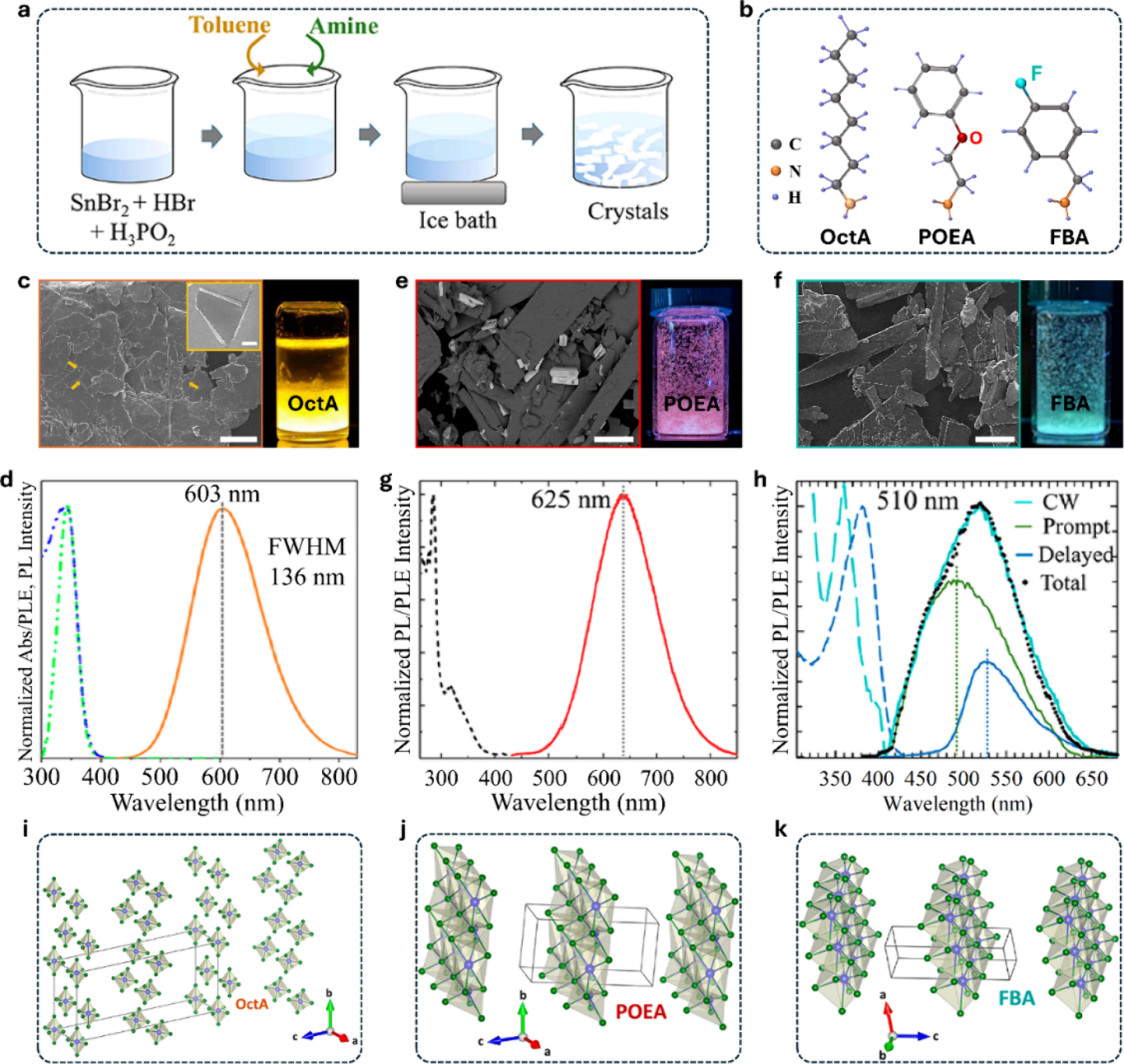
**a, b**, Schematic of the low-temperature synthesis of Sn–Br RP-related layered perovskites with different organic cations. **c**, SEM image of OctA-based platelets. Scale bar: 100 μm. Inset: magnified view of the platelet edges, highlighting the rolled edges from the thin nature of the crystals. Scale bar: 5 μm. Right: photographs of the crystals under UV light. **d**, Absorption (blue), PLE (green), and PL (orange) spectra of OctA-based crystals. **e, f**, SEM images of POEA- and FBA-based Sn–Br RP-related layered perovskites. Scale bar: 100 μm. Right: photographs of the crystals under UV light. **g, h**, PLE (dashed) and PL (solid/dotted) spectra of POEA- and FBA-based crystals. In (h), time-gated (green), delayed (blue), and summed (black dotted) PL. **i-k**, Crystal structures of the inorganic layers in OctA-, POEA- and FBA-based Sn–Br RP-related layered perovskites from 3D ED analysis. Adapted from reference^12^. Copyright 2023 American Chemical Society.

To explore organic cation effects, we selected 2-phenoxyethylammonium (POEA) and 4-fluorobenzylammonium (FBA) cations, where heteroatoms introduce local dipoles and charge separation. The resulting crystals exhibited elongated platelet-like morphologies (Figure 6e, f), with strongly Stokes-shifted orange-red broadband emission with a PLQY of ∼15% from the POEA-based crystals and blue-green broadband emission from the FBA-based structures (Figure 6g-h). These cation-dependent interactions distort the Sn–Br octahedra (Figure 6i-k) and open distinct STE relaxation pathways, providing a rational route to tunable emission in Pb-free systems. Under continuous-wave laser excitation, the FBA-based crystals exhibited delayed fluorescence, suggesting the presence of nonradiative trap states that facilitate slow carrier release. Both systems remained structurally and optically stable for approximately seven months under ambient conditions.

In Sn-based layered perovskites, emission can arise from band-edge free excitons as well as intrinsic and extrinsic trapped states, including defect-assisted trapping associated with exciton-defect scattering centers. Analogous to Pb-based systems, organic cations not only influence structural distortions, inorganic–organic interactions, and exciton–phonon coupling, but also modulate the density and nature of defect states in Sn-based materials. For example, exciton relaxation is often governed by deformation-potential scattering from charged defects, which can directly impact optical efficiency.^40^ Furthermore, halide composition and precursor stoichiometry, along with additives that coordinate Sn^2+^ and strengthen hydrogen-bonding interactions, provide additional levers to tune emission pathways, suppress ion migration, and improve stability.^45^

Overall, these findings highlight the versatility of RP layered perovskites and demonstrate how subtle changes in the molecular structure of organic cations can drive pronounced structural and optical transformations, providing a promising framework for other Pb-free systems. Moreover, combining molecular design with straightforward synthetic approaches opens opportunities for high-throughput and AI-driven materials discovery, enabling the screening and identification of cost-effective and efficient materials for targeted applications.^26^

An emerging frontier in 2D organic–inorganic layered perovskites is the substitution of divalent metal cations by an ordered combination of monovalent and trivalent metal cations (e.g., Ag^+^, Bi^3+^, Cu^+^, In^3+^), arranged in a rock-salt configuration,^46^ forming the so-called double-layered perovskites. This cation ordering not only stabilizes the perovskite framework but also expands the compositional space, enabling access to new structural motifs and potentially new physical and optical properties. In their 3D analogues, STEs induced by Jahn–Teller distortions in the metal-halide octahedra can yield efficient and stable white light emission.^47^ In contrast, experimental demonstration of 2D double-layered perovskites remains limited, and it is often hindered by synthetic challenges and phase stability due to the structural complexity, arising from alternating metal cation layers separated by organic cations.

### Elemental Doping

In addition to complete metal substitution, the optoelectronic properties of RP layered perovskites can be further tailored through elemental doping with extrinsic divalent metal cations, providing a route for emission engineering without fully altering the host lattice. Among the various dopants explored, Mn^2+^ is particularly effective, enabling emission tunability across the visible spectrum through the coexistence of band-edge excitonic emission and a large Stokes-shifted, broadband red-orange PL originating from the intraionic ^4^T_1_ → ^6^A_1_ transitions of Mn^2+^ (Figure 7a). The spectral position and efficiency of Mn^2+^-related emission are highly sensitive to dopant concentration and local coordination, highlighting the need for precise synthetic control.

**Figure 7:**
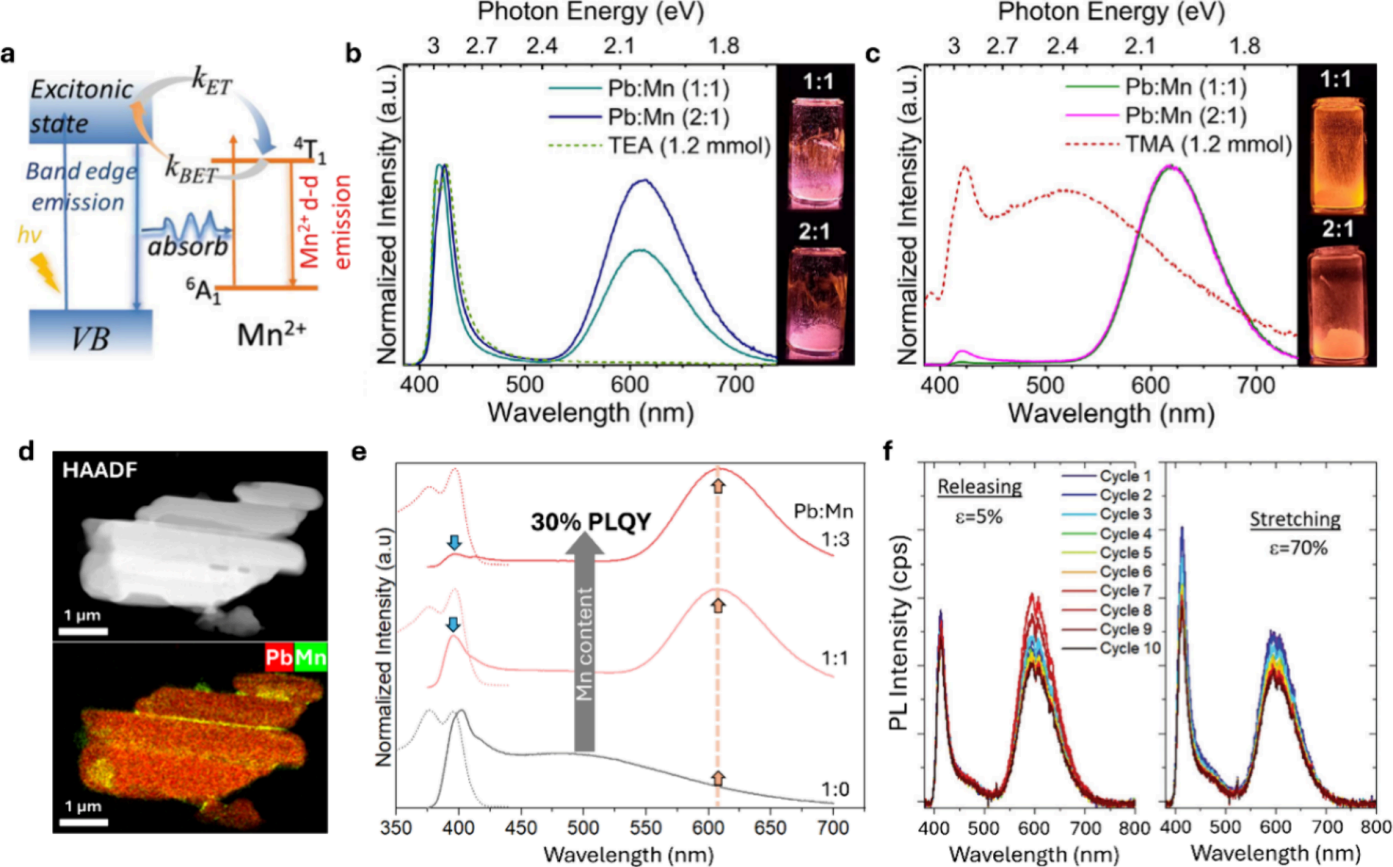
**a**, Energy-level scheme of Mn-doped RP layered perovskites. Reproduced with permission from reference^48^. Copyright 2019 Royal Society of Chemistry. **b-c**, PL spectra of Mn-doped TEA- (b) and TMA-based (c) RP Pb–Br layered perovskites at varying Mn content. Adapted from reference^34^. Copyright 2021 Wiley-VCH. **d**, HAADF-STEM and corresponding Pb/Mn elemental map of grooved BzA-based crystals. **e**, Absorption (dotted lines) and PL (solid lines) spectra of Mn-doped grooved crystals at different Pb:Mn ratios. Adapted from reference^11^. Copyright 2025 Wiley-VCH. **f**, Optomechanical response of Mn-doped polymer composite films under cyclic stress. Adapted from reference^20^. Copyright 2021 Royal Society of Chemistry.

The choice of organic cation strongly influences the relative contribution of the perovskite’s band-edge and Mn^2+^-centered emissions, independent of the Mn doping level. For example, Mn-doped TMA-based crystals exhibited predominantly Mn-related broadband emission, whereas Mn-doped TEA-based crystals displayed dual emission consisting of comparable band-edge and Mn^2+^-centered orange-red emission components (Figure 7b, c),^34^ as observed in PEA.^48^ Beyond homogeneous bulk doping, structural anisotropy can be exploited to achieve spatially selected dopant incorporation. We recently demonstrated a preferential substitution of Pb^2+^ to Mn^2+^ through postsynthetic Mn-doping at groove boundaries and facet edges (Figure 7d) of the elongated structures prepared with BzA and discussed earlier.^11^ This spatial selectivity is driven by surface energy minimization, as the disconnected ribbons inherently provide numerous high-energy internal edge sites for dopant incorporation, in a similar fashion as in BA-based RP perovskites, where excitons are located in the edge and interior of the inorganic layer.^49^ As a consequence of this facet-selective doping, the emission of RP crystals can be tuned from white to pink-orange, with an enhanced PLQY up to ∼ 30% in highly anisotropic structures (compared to ∼ 18% for the pristine host) (Figure 7e). These results illustrate how localized doping may enable spectral tunability within a single structure, and while pointing toward future opportunities for nanoscale patterning, directional energy transfer, and spatially resolved photocatalysis, detailed investigation of dopant distributions and exciton transport is still required to fully understand and exploit such anisotropic layered structures.

The versatility of Mn-doping in layered perovskites has also been demonstrated in polymer composite films using in situ BzA and PEA organic cations.^20,50^ These hybrid films exhibited dual emission, a band-edge PL centered in the blue region and a broader Mn^2+^ intraionic transition around 610 nm. Under continuous cyclic mechanical stress, the films displayed enhanced blue emission due to reduced reabsorption and altered in-plane and out-of-plane dipole orientations caused by the relative sliding and reorientation of perovskite platelets (as discussed in the previous section), while maintaining the red Mn^2+^ emission intensity (Figure 7f).

Altogether, these developments emphasize the chemical versatility of 2D RP layered perovskites as an adaptable platform for next-generation light-emitting materials. Through the synergistic application of (i) organic cation design, (ii) compositional tuning, and (iii) controlled doping chemistry, precise regulation of optoelectronic properties becomes accessible. Continued exploration of Pb-free layered perovskites and alternative dopants will be essential to translate these materials into sustainable optoelectronic and photonic technologies.

## 4 Conclusions and Outlook

The interplay between organic and inorganic layers in 2D layered perovskites offers a rich compositional and structural platform for tailoring optoelectronic properties. Their solution processability, compatibility with low-temperature synthesis, and structural versatility position layered perovskites as promising candidates for low-cost, high-performance light-emitting technologies. In this Account, we have shown that organic-cation engineering and solvent-cation interactions act as powerful structure-directing strategies in RP layered perovskites, enabling control over octahedral distortions, lattice dynamics, exciton–phonon coupling, and defect landscapes. Through these mechanisms, emission can be modulated between free-exciton- and STE-dominated regimes.

We further show that these design principles extend to Pb-free analogues, where suppressing defect-assisted recombination and metal oxidation requires deliberate coupling of organic cation design with inorganic compositional engineering. Metal substitution and controlled elemental doping provide complementary routes to tune band structure, regulate lattice softness, and engineer radiative pathways. Together, these results highlight three key insights:

i) Organic cation rigidity and packing regulate exciton–phonon coupling and nonradiative decay;
(ii) Solvent-mediated structural reconfiguration provides a practical approach to modulate octahedral connectivity and emission pathways; and
(iii) Engineered motifs such as disconnected ribbons and grooved facets create chemically addressable sites for localized doping and emission control.

Despite this progress, significant technological challenges remain. A central objective is the development of quantitative structure–property descriptors capable of predicting emission behavior, particularly for stable white-light generation. While empirical strategies have enabled substantial advances, predictive design frameworks that correlate molecular descriptors, such as steric profile, dipole moment, hydrogen-bonding motifs, and conformational flexibility with exciton binding energy, self-trapping propensity, defect densities, and operational stability are still underdeveloped. Establishing such correlations will be critical for transitioning from exploratory synthesis to rational materials engineering.

At the device level, purely 2D (*n* = 1) RP layered perovskites continue to face limitations, including inefficient charge injection across organic-rich barriers, ion migration, and challenges in controlling thin-film orientation and phase purity. Although quasi-2D phase mixtures have enabled improved external quantum efficiencies (EQE) through exciton funneling,^51^ systematic control over phase distribution and operational stability are still open challenges.

Similar constraints apply to Pb-free systems, with reported EQEs around 1% for 4-bromobenzylammonium-based Sn–Br LEDs,^42^ and up to 20% for TEA-based Sn–I devices.^43^ Even when elemental doping increases PLQY, device EQEs often remain modest,^52^ highlighting the need for advances in interface engineering, defect passivation, and charge transport layer optimization.

Looking forward, expanding the chemical toolbox of organic cations offers a substantial opportunity. Beyond conventional organoammonium cations, alternative ligands, such as sulfur-, phosphonium-, and carboxylate-based motifs, may enable strong binding and improved lattice stabilization, as well as ligands that template low-dimensional Pb-halide lattices and yield stable white-light emission.^53,54^ Such chemistries could facilitate access to new structural phases and emergent photophysical behavior not achievable within the current design space.

Another promising direction lies in heterostructures. Organic-templated heterostructures^55^ formed within the van der Waals gaps of RP layered perovskites and mixed-dimensional heterostructures provide opportunities to engineer band alignment and extend spectral coverage toward the near-infrared (NIR).^56,57^ Integrating environmentally benign NIR emitters within layered perovskite frameworks may broaden the accessible emission range, preserving structural tunability.

Finally, the integration of AI, robotics, and high-throughput experimentation in self-driven laboratories is poised to transform layered perovskite discovery.^58^ Given the high-dimensional design space defined by organic chemistry, processing conditions, and inorganic composition, AI-assisted approaches can accelerate the identification of stable and efficient structures. However, the application of data-driven methods in this field is often challenged by limited and heterogeneous data sets.

Emerging workflows combine high-throughput experimentation with machine-learning models trained on chemically informed descriptors, including physicochemical, steric, and topological descriptors, as well as solvent and processing variables. Such approaches have enabled, for example, the identification of viable organic cations for 2D Ag/Bi-iodide perovskites, even in data-sparse regimes.^59^ Integrating AI-driven workflows with mechanism-guided design principles outlined in this *Account* offers a powerful route to predictive layered perovskite design that fulfills the *Triple E* paradigm and supports next-generation lighting and photonic technologies.
